# WHO recommended collaborative TB/HIV activities: evaluation of implementation and performance in a rural district hospital in Cameroon

**Published:** 2011-11-02

**Authors:** Habakkuk Azinyui Yumo, Christopher Kuaban, Florian Neuhann

**Affiliations:** 1 National AIDS Control Committee, Ministry of Public Health, Yaoundé, Cameroon; 2 Faculty of Medicine and Biomedical Sciences, University of Yaoundé I, Yaoundé, Cameroon; 3 Jamot Hospital, Yaoundé, Cameroon; 4 Institute of Public Health, University of Heidelberg, Heidelberg, Germany

**Keywords:** Implementation, performance, tuberculosis, HIV, rural, district, health facility, Cameroon

## Abstract

**Background:**

The objective of the study was to assess the implementation and the performance of recommended collaborative TB/HIV activities in Batibo District Hospital (BDH) and to determine the prevalence of HIV in TB patients in this rural locality.

**Methods:**

The implementation of collaborative TB/HIV activities was assessed through interviews with health workers in the hospital. The implementation score was calculated as the proportion of recommended activities effectively implemented in the hospital. The performance of implemented activities and the prevalence of HIV were determined through review in HIV and TB registers of routine data for the period 2003-2008.

**Results:**

The implementation of collaborative TB/HIV activities though triggered by the existence of both TB and HIV units in the hospital was only moderate with an implementation score of 50%. All implemented activities aimed at reducing the burden of HIV in TB patients. The performance of implemented activities was in average 61% (n=179) and 82% (n=77) respectively regarding HIV testing among TB patients and antiretroviral therapy coverage in TB/HIV co-infected patients. Provision of isoniazid preventive therapy (IPT) was inexistent in this hospital due mainly to the lack of tuberculin skin test and isoniazid tablets. The prevalence of HIV among TB patients in this rural locality was 53%. This prevalence was 55% in females and 44% in males (p=0.19).

**Conclusion:**

The implementation of collaborative TB/HIV activities in BDH was effective only regarding activities to reduce the burden of HIV among TB patients. There is urgent need to strengthen the capacity of this rural health facility in providing IPT services.

## Background

The human immunodeficiency virus (HIV) epidemic has led to the reemergence of tuberculosis (TB) worldwide and more particularly in countries with high HIV prevalence [1]. The HIV infection increases the risk of TB reactivation [2–5], causes rapid progression to active TB disease, and increases the risk of dying during a TB episode [6]. TB remains the most common opportunistic infection for people living with HIV, including those on antiretroviral therapy, and a leading cause of death for people living with HIV, especially in low and middle income countries [7]. In 2009 globally, approximately 24 % of patients who died from TB were HIV co-infected [8].

The impact of the TB/HIV cohabitation in the epidemiology and the clinical outcome of both diseases are of important programmatic concerns to TB and HIV programs and implications for policies include the need to promote TB and HIV/AIDS program collaboration [9] both at strategic and facility level. Actually, the strong relation between HIV and TB morbidity in high prevalence countries had stimulated debates and eventually influenced policy about integration of TB and HIV services. Nonetheless, the integration of these programs faces challenges all the way from policy making to integration at facility level [10]. In 2005, describing some of these challenges in the Democratic Republic of Congo, Martinot reported that some HIV activities were integrated only in 58% of TB clinics surveyed [11].

In response to this need of integrating TB/HIV activities the World Health Organization (WHO) in 2004 mapped out 12 key activities to be implemented by countries to ensure effective collaboration between TB and HIV programs [12]. From these key activities a set of core indicators was defined for monitoring and evaluating collaborative TB/HIV activities [13].

According to UNAIDS, Cameroon has an HIV prevalence of 5.3% [14]. This relatively high HIV prevalence has undoubtedly contributed to the resurgence of TB and the growing burden of this disease in recent years in this country. Here, the estimated TB incidence (all forms) has risen from 81 (per 100 000) in 1990 to 182 in 2009 and the prevalence of HIV among TB patients was 40% in 2008 [8]. In 2006, Cameroon was among the 63 TB/HIV priority countries expected to commence the implementation of all recommended collaborative TB/HIV activities [15]. In line with this recommendation, TB and HIV management guidelines of this country indicate the implementation of collaborative TB/HIV activities at facility level, including among others tuberculin skin test (TST) prior to IPT provision [16,17]. The Batibo District Hospital (BDH) is such an operational site. The aim of this study was to assess the implementation and performance of this hospital regarding these activities. It was also aimed at determining the prevalence of HIV in TB patients in this rural health setting. This evaluation is important because it will inform implementers and program managers on the successes and challenges related to the expansion of TB/HIV activities in rural areas.

## Methods

### Setting

The BDH is the district hospital of the Batibo Health District consisting of 13 health areas. Located in a rural area in the North West Region of Cameroon, this public hospital caters for a population of 101 576 and has a capacity of 86 beds. It is the first referral hospital for the 19 satellite health centers of the health district and provides a range of polyvalent health care to the population. The hospital has a functional center for diagnosis and treatment of TB (CDT) since October 2002. A passive case detection strategy is used to identify TB cases. All diagnosed TB cases are initiated on standard TB regimens, followed up and outcomes are reported according to national TB control guidelines [16]. The hospital also has a functional HIV/AIDS management unit since September 2005. This unit provides VCT, HIV treatment, care and support to the population of the health district and the surrounding areas. Integration of TB/HIV services in this hospital started in 2005 with the creation of the HIV/AIDS management.

### Design

To evaluate the implementation and the performance of the collaborative TB/HIV activities, we carried out a cross sectional study in BDH in July 2009. The evaluation criteria were the WHO recommended TB/HIV activities and related core indicators, representing the performance of implemented activities were calculated as presented in additional material 1 [13]. To determine the prevalence of HIV in TB patients, records of all patients treated in the hospital for TB from 2003-2008 were reviewed.

### Data collection

Data on the implementation of collaborative TB/HIV activities were collected through interviews with medical (1 medical doctor) and para-medical (3 nurses and 2 counselors) staff involved in TB and/or HIV programs in the hospital to determine enabling factors and obstacles for collaborative TB/HIV activities at facility level. In this study, an activity was qualified as implemented when it was delivered in the hospital as per reported by staff or registers. Data on the performance of implemented activities and the prevalence of HIV were determined through review of registers (HIV and TB) as well as drugs stocks cards for routine programmatic data for the period 2003-2008. Actually, a structured questionnaire was used to extract data related to core indicators of collaborative TB/HIV activities as well as the HIV sero-status of patients treated in the hospital. For each TB patient, we collected socio-demographic data (age, sex, residence) and information related to HIV, ART and cotrimoxazole prophylaxis therapy (CPT) status. For each HIV patient we collected information related to the screening of latent TB and Isoniazid Preventive Therapy (IPT).

### Statistical Analysis

The implementation level of the hospital with regards to the WHO-recommended collaborative TB/HIV activities was determined as the implementation score of the recommended activities. This score was a rate calculated using as numerator the total number of recommended activities effectively implemented in the hospital and as denominator the total number of recommended activities for the operational level. The performance of implemented activities was represented by the related core indicators. The core indicators assessed were the following: TB patients tested for HIV, HIV patients screened for TB, TB/HIV patients on ART (also representing the level of HIV care and support), TB/HIV patients on cotrimoxazole Preventive Therapy (CPT) and HIV patients on IPT. These indicators were calculated as the proportions of patients who benefited from the related services. For the analysis of these core indicators as well as the HIV sero-status of TB patients, data were entered and analyzed using Epi Info (version 3.5.1, CDC, Atlanta). The core indicators and prevalence were expressed in percentages. Chi-square (X2) test, with a significant level at 5% was used to compare frequencies.

### Ethical Clearance

This study was a desk review analysis without contact with human subjects. The research protocol was approved by the ethics committee of the University of Heidelberg and the Batibo District Hospital administration authorized the conduct.

## Results

A total of 293 patients were enrolled on TB treatment from 2003 to 2008. 54% (158) were males. The age ranged from 3 to 63 years (median=31 years). Children below 15 years of age represented 2.7% (N= 8) of the cohort while the age group 25-44 accounted for 57.7% (N=169) of the study population. From 2005 to 2008 the HIV unit initiated 638 people living with HIV/AIDS on ART. Additional material 2 depicts the status of the implementation of collaborative TB/HIV activities in BHD and factors enabling or hindering the implementation of these activities.

Additional material 1 shows that of the 8 WHO recommended collaborative TB/HIV activities implementable at facility level, only 4 were being implemented in BDH at the time of this review, giving an implementation score of 50%. The analysis of staff interviews shows that the non-implementation of IPT activities was attributed to the lack of technical skills of staff in providing TST and IPT services coupled with the unavailability of TST kits and INH in the hospital. The implementation of HIV testing among TB patients with provision of ART to eligible cases was boosted by the availability and affordability of HIV tests and ARV drugs as the result of the creation of the HIV unit in hospital in 2005.

**Figure 1:**
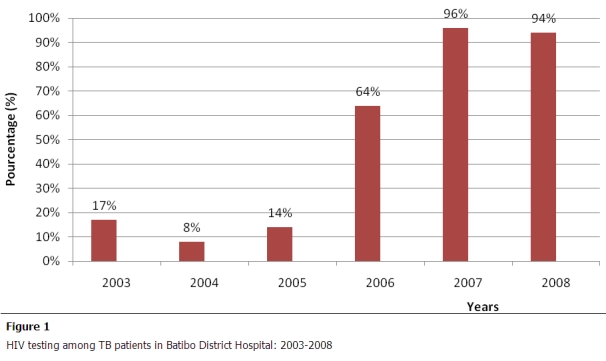
HIV testing among TB patients in Batibo District Hospital: 2003–2008

**Figure 2:**
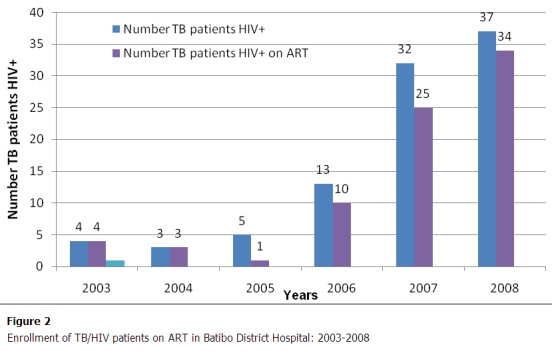
Enrollment of TB/HIV patients on ART in Batibo District Hospital: 2003–2008

From the 293 TB patients analyzed, 61% (179/293) were tested for HIV and of those 99% (177/179) had a conclusive HIV test result. This HIV testing rate among TB patients varied across years from 17% in 2003 to 94% in 2008, with a pick at 96% in 2007 (Figure 1). Of the 177 persons having a conclusive HIV test result, 94 were positive for HIV, giving a prevalence rate of 53% (95%CI: 45- 60). This prevalence was 55% (95% CI: 44- 65) in females as compared to 44% (95%CI: 34-55) in males with no significant statistical difference between the two sexes (p=0.19). The age of HIV positive TB cases ranged from 17 to 60 years with a mean age of31±8 and 35±9 years respectively for females and males. From the 94 TB/HIV co-infected patients, 78 (83%) did a CD4 count. From this number, 77 (82%) were enrolled on ART. Enrolment on ART varied across years to a maximum rate of 92% (34/37) in 2008 (Figure 2).The ART coverage rate also represents the level of HIV care and support to TB/HIV patients. It was not possible to calculate the core indicator related to the implementation of CPT because though delivery of free cotrimoxazole was effective in hospital, routine data on this activity was inaccurate due to inconsistent reporting in the registers. Furthermore, no HIV patient was on IPT in the hospital at the time of the review. Table 1 summarizes the core indicators (performance) of collaboration TB/HIV activities in the hospital for the study period.

**Table 1:** Summary table of performance indicators of WHO collaborative TB/HIV activities in Batibo District Hospital

No	Core indicators	Performance of implemented TB/HIV activities		
		
		numerator	denominator	Value (%)
**Activities to reduce the burden of HIV in people infected with TB**				
**1**	Proportion of TB patients tested for HIV	179	293	61
**2**	Proportion of TB/HIV co-infected patients enrolled in pre-ART register or ART register once started on ART during TB treatment (HIV care and support)	77	94	82
**3**	Proportion of TB/HIV co-infected patients initiated on ART	77	94	82
**4**	Proportion of TB/HIV co-infected patients started on or continued on previously initiated CPT during TB treatment	Data not available^*^		
**5**	Availability and distribution of free condoms at the TB clinic	No		
**Activities to reduce the burden of TB in people living with HIV (The 3Is)**				
**6**	Proportion of adults and children enrolled in HIV care whose TB status were assessed and recorded during their last visit.	0	638	0
**7**	Proportion of adults and children newly enrolled on HIV care who are started on treatment for latent TB infection (are given at least one dose of IPT) during the reporting period.	0	638	0
**8**	Practice of TB control measures in the hospital	No		

* CPT is implemented, but reporting is inconsistent

## Discussion

This evaluation has shown that, with an implementation score of 50%, the implementation of collaborative TB/HIV activities in BDH is only moderate. Not implementing the so called 3Is activities in the hospital has contributed for 75% in this result. Actually, screening of HIV patients for TB infection and provision of IPT to eligible cases is not done in BDH. This service gap is not specific to this hospital, but goes across HIV treatment centers (HTCs) in Cameroon. Indeed, countrywide less than 10% of HTCs provide IPT services to HIV infected patients with latent TB infection [18]. Like argued by Harries et al. [19], this situation in BDH is related to the technical (lack of skills) and logistical (lack of test kits) challenges hindering the use of tuberculin skin test (TST) as well as the fear of care providers to initiate in IPT patients in whom active tuberculosis cannot be completely excluded. In BDH, these challenges are compounded by the unavailability of Isoniazid (INH) for IPT in the hospital. This lack of implementation stands in sharp contrast to the compelling evidence on the effectiveness of IPT in reducing the risk of tuberculosis in HIV patients [19]. It has been reported still by Harries that one of the reasons for the non implementation of IPT in countries is that at program level, it is not clear as to who assumes the responsibility for planning and implementing IPT activities [20]. Should it be the TB or the HIV program? According to the WHO the implementation of IPT services is the responsibility of the National HIV program [13]. However, this organization recommends the setting up at central level of a coordinating body for collaborative TB/HIV activities with the mandate to: develop joint strategic plans, mobilize resources, build capacity, implement and monitor collaborative TB/HIV activities. This coordination body should comprise of experts from the national HIV and TB programs, and other partners working in this field of co-infection TB/HIV. The absence of this body in a country may jeopardize national implementation of such activities [13]. This body was recently created (October 2009) in Cameroon. This may also explain the weaknesses observed in the implementation of IPT services in the country during the study period. Four out of 5 (80%) of the recommended activities to reduce the burden of HIV among TB patients were being implemented in BDH. Only 1 (one) of these activities was lacking. This missing activity was HIV prevention among TB patients, notably the provision of free condoms to patients at TB unit. Free condoms provision to the population, including to HIV or TB patients is not a policy in Cameroon. Instead condoms are made affordable to the populations through a subsidized cost of $US 0.25 per unit (4 condoms). Therefore, in addition to other HIV prevention methods, staff at TB clinics should emphasize on systematic counseling of TB patients in practicing safe sex, notably condom use.

HIV testing among TB patients increased remarkably in 2005. This improvement coincided in time with the opening of the HIV/AIDS unit in the hospital in 2005. This improved performance can be explained by the integration of TB/HIV services in the same facility. In BDH, this integration boosted the morale of health workers in requesting HIV tests from patients, while increasing the willingness of patients in accepting HIV testing. Indeed, 93% of HIV testing conducted among TB patients was during the period 2006-2008, corresponding to the period after the creation of the HIV unit in the hospital. This very good rate needs though to been seen in relation to the low detection rate for TB during the evaluation period, but also clearly underlines the advantages of integrating affordable TB/HIV services in the same health facility as highlighted by Yumo et al.(2011) in another study still in Batibo District Hospital [21]. It is worth to remark that the high HIV testing uptake recorded in BDH among TB patients is also attributable to the provider-initiated HIV testing and counseling (PITC) (or opt-out-approach) introduced in the hospital in 2007. As compared to the opt-in-approach used in hospital before 2007, PITC is more effective in increasing HIV testing uptake, and most importantly it is feasible even rural health settings as previously reported in BDH [22] and elsewhere [23]. With an HIV testing rate of 94% in 2008, the target of the Global Plan 2006-2015 of STOP TB to HIV-test and counsel at least 85% of TB patients in DOTS programs [13]; is already achieved in Batibo Health District. Continuous efforts of programs managers at all levels are needed to maintain this performance by 2015. In particular, managers should ensure regular supply of HIV testing kits to the facility in a bid to avoid stock outs that may disrupt this already achieved performance.

The proportion of TB/HIV co-infected patients enrolled on ART was 82% (n=77). This ART coverage rate is higher than the 36% observed at national level in 2008[8]. This high performance is attributed to the strong link between the TB and HIV units of the hospital. Here TB patients are systematically referred by the TB/HIV counselor to the HIV unit for HIV testing. Cases diagnosed with HIV infection are further investigated for ART eligibility; eligible cases initiated on ART and followed up according to the national guidelines [17]. It is interesting to observe that only 25% (3/12) of eligible cases were enrolled on ART during the period (2003-2005) before the creation of the HIV unit as compared to 90% (74/82) during the period (2006-2008) after the creation of this unit. This finding clearly demonstrates the effectiveness of having both TB and HIV units in the same health facility as a strategy to speed up ART coverage among TB/HIV co-infected patients and further supports the need for integrating TB/HIV services as mentioned above.

CPT coverage rate could not be calculated because TB registers at the TB unit had no provision to track CPT status. On the other hand, cotrimoxazole registers at the HIV unit was inconsistently filled and more so had no provision to track the TB status of HIV patients on CPT. CPT and even ART reporting gap at global level was already outlined by Gunnerberg et al. (2008) who found that only 1/3 of countries was reporting data on CPT and ART among TB/HIV co-infected patients [24]. To ensure effective monitoring and evaluation of collaborative TB/HIV activities, routine data collection registers should include the following specifications: 1) The HIV register should have provision to track TB co-infection, TB treatment, IPT and CPT status of patients; 2) TB registers should have provision to track HIV co-infection, ART and CPT; and 3) cotrimoxazole registers should have provision to track TB co-infection, ART, INH, TB treatment. Furthermore, implementation of CPT in BDH was found to be hampered by recurrent stock outs of free cotrimoxazole due to lack of supply from the central level. This weakness needs to be appropriately addressed so as to enable TB/HIV co-infected patients on care in this hospital benefiting from CPT effect in mortality reduction [25].

HIV prevalence (53%) among TB patients in BDH was higher than the 40% reported by WHO for the national level in 2008 [8], but similar to the 52% reported in a urban district hospital in Douala (Cameroon) still in 2008 by Sume et al. [26]. This finding suggests that, in contrast to the general population where HIV prevalence is significantly higher in the cities as compared to the rural areas [27], the burden of HIV infection among the subpopulation of TB patients may be similar between cities and rural localities. Nevertheless, this high HIV prevalence among TB patients in BDH should be interpreted with caution since it may have been affected by a selection bias. Indeed, migration of Batibo natives from the cities to seek care in BDH is common. Secondly, Batibo is a transit zone in the trans-african (Cameroon-Nigeria) highway with occasional influx of outsiders. It is possible with these phenomena that the prevalence of HIV be higher in Batibo Health District as compared to typical rural areas. The TB/HIV co-infection rate, though not statistically significant was higher in females. This result parallels previous studies in Cameroon [24,28] and further supports evidence of the feminization of HIV infection in Cameroon [27].

This study has some limitations. The data was collected through staff interviews and registers reviews. These methods may have introduced some information biases either from the staff or inaccurate reporting in the registers. Nevertheless, the TB and HIV/AIDS programs were funded by the Global Fund (GF) as from 2005 [21]. Considering the data management exigencies of GF funded programs, these biases if they exist should be minimal.

## Conclusion

In conclusion, the implementation of collaborative TB/HIV activities in BDH was only moderate due mainly to the non implementation of IPT activities despite the existence of both TB and HIV units in the hospital. The performance of implemented activities was good regarding HIV testing among TB patients and excellent concerning HIV care and support to TB/HIV co-infected. The prevalence of HIV in TB patients was high in BDH. Strengthening the capacity of the BDH in the 3Is activities and particularly in IPT services is urgently needed to achieve complete collaborative TB/HIV activities in this rural health facility. Addressing this gap in IPT provision should be easier now since according to the 2010 WHO Stop TB recommendation, TST is not more required to initiate HIV patients on IPT [29].

## Additional material

### Additional material 1

WHO collaborative TB/HIV activities and related core indicators recommended for facility level: http://www.panafrican-med-journal.com/content/article/10/30/material/10-30_1.pdf (PDF 52Kb)

### Additional material 2

Status of implementation of WHO recommended collaborative TB/HIV activities in BDH. July 2009: http://www.panafrican-med-journal.com/content/article/10/30/material/10-30_2.pdf (PDF 47Kb)
